# A phase II irinotecan–cisplatin combination in advanced pancreatic cancer

**DOI:** 10.1038/sj.bjc.6601377

**Published:** 2003-11-11

**Authors:** C Markham, D D Stocken, A B Hassan

**Affiliations:** 1 Liver Unit, University Hospital Birmingham NHS Trust (Queen Elizabeth), UK; 2 Cancer Research UK Clinical Trials Unit and Institute for Cancer Studies, University of Birmingham B15 2TT, UK; 3 Bristol Haematology and Oncology Centre, Horfield Road, Bristol BS2 8ED, UK

**Keywords:** pancreatic cancer, irinotecan, cisplatin, palliative chemotherapy, patient selection

## Abstract

We report a cisplatin and irinotecan combination in patients with biopsy-proven advanced pancreatic adenocarcinoma. Patients were selected from a specialist centre and required good performance status (KPS>70%), measurable disease on CT scan, and biochemical and haematological parameters within normal limits. Based on a two-stage phase II design, we aimed to treat 22 patients initially. The study was stopped because of the death of the 19th patient during the first treatment cycle, with neutropenic sepsis and multiorgan failure. A total of 89 treatments were administered to 17 patients. Serious grade 3/4 toxicities were haematological (neutropenia) 6%, diarrhoea 6%, nausea 7% and vomiting 6%. Using the clinical benefit response (CBR) criteria, no patients had an overall CBR. For responses confirmed by CT examination, there was one partial response (5%), three stable diseases lasting greater than 6 weeks (16%), with an overall 22% with disease control (PR+SD). The median progression-free and overall survival was 3.1 months (95% CI: 1.3–3.7) and 5.0 (95% CI: 3.9–10.1) months, respectively. Although this synergistic combination has improved the response rates and survival of other solid tumours, we recommend caution when using this combination in the palliation of advanced pancreatic cancer, because of unexpected toxicity.

Once diagnosed, patients with pancreatic adenocarcinoma have an average life expectancy of 16–20 weeks. Clinical management has an emphasis on palliative support because of the poor prognosis and the rapidly deteriorating quality of life due to the syndrome of fatigue, weight loss, pain and jaundice (Wigmore *et al*, 1997; Andreyev *et al*, 1998). Standard selection criteria, as used for most other solid tumours, can often exclude a significant proportion of pancreatic cancer patients, with trials reporting results in selected patients with good performance status. Toxic treatments that follow can lead to early withdrawal from studies, may worsen otherwise the good quality of life and, in some instances, shorten the duration of life. The use of low-toxicity agents, such as gemcitabine and metalloproteinase inhibition, have had notable success in terms of trial recruitment, patient compliance and treatment tolerance (Carmichael *et al*, 1996; Burris *et al*, 1997; Bramhall *et al*, 2001).

The 5-year survival rate for pancreatic cancer remains at 2% (Bramhall *et al*, 1995). Single-agent chemotherapy, such as 5-flurouracil, paclitaxel and gemcitabine, all result in radiological response rates between 5 and 15% (Burris *et al*, 1997; Whitehead *et al*, 1997). Combination chemotherapy, including drugs such as cisplatin, 5-FU, adriamycin and gemcitabine, have generally improved the response rates slightly, at the expense of increasing toxicity in some combinations (Cascinu *et al*, 1996; Evans *et al*, 1996; Hidalgo *et al*, 1999). Aside from the differences in patient selection, one problem with the interpretation of these studies is the reliability of radiological response, mainly because of the dense fibrotic reaction that often occurs within pancreatic tumours (Ahlgren, 1996). As a result, survival data are often quoted in combination with surrogate factors, for example, the clinical benefit response (CBR). The latter incorporates a scoring system for positive and negative changes in pain, performance status and weight, and has been an important tool in establishing gemcitabine efficacy (Rothenberg *et al*, 1996a).

Here we report the activity and toxicity of the drug combination, irinotecan and cisplatin, in previously untreated patients with advanced pancreatic cancer. This combination has been shown to generate significant short-term radiological response rates and improvement in survival in solid tumours, most notably in small-cell lung cancer (Noda *et al*, 2002, Ilson *et al*, 2003; Souid *et al*, 2003).

Irinotecan is a camptothecin analogue and topoisomerase I inhibitor with a highly active metabolite (Sn38). This agent has demonstrated improved survival in metastatic 5-FU refractory colorectal cancer (Cunningham and Glimelius, 1999; Rothenberg *et al*, 1999). Laboratory studies show high response rates of pancreatic tumour cells in culture and in xenograft studies (Takeda *et al*, 1992; Bissery *et al*, 1996). Single-agent phase II studies of irinotecan in pancreatic carcinoma (dose intensity 100 mg m^−2^ week^−1^) have shown typical response rates of around 10%, again similar to other single agents (Wagener *et al*, 1995; Armand *et al*, 1996). There are now data showing significant synergy between cisplatin and irinotecan in lung cancer cell lines *in vitro* (Kanzawa *et al*, 2001). However, there are no published data concerning cisplatin and irinotecan alone in advanced pancreatic cancer, although other agents have been successfully combined with irinotecan in this disease, for example, gemcitabine (de Jonge *et al*, 2000; Kozuch *et al*, 2001; Rocha Lima *et al*, 2002; Slater *et al*, 2002).

## MATERIAL AND METHODS

### Patient selection and study design

Eligible patients were chemotherapy naïve (>18 years), and had pancreatic adenocarcinoma diagnosed by histology with measurable disease on CT scan. Karnofsky performance status (KPS)>70%, either stent insertion or hepato-jejunostomy for biliary drainage, bilirubin <1.5 × upper limit of the normal (<35 *μ*mol l^−1^), AST <5 × upper limit of the normal, GFR >60 ml min^−1^ based on Cockcroft formula and confirmed by creatinine clearance in borderline cases, neutrophils >1.5 × 10^9^ l^−1^ and normal blood count profile with no clinical history of inflammatory bowel disease or previous malignancy (except non-melanoma skin cancer and *in situ* cervical carcinoma). The study was approved by the South Birmingham Local Ethics Committee and all patients gave written informed consent. All patients were requested to complete a pain inventory of all analgesic medication, and pain was assessed using the Wisconsin brief pain questionnaire and visual analogue scale (assessed every evening at the same time).

The end points of this study were the radiological response rate (CR+PR), disease control (CR+PR+SD), overall survival (defined as the time from entry into the trial to the date of death or censor), progression-free survival (PFS) at 3 months (defined as the time from entry into the trial to the first objective documentation of progression), CBR and toxicity. Clinical benefit response was assessed as recommended by Rothenberg *et al* (1996b). In summary, primary measures were defined as >20% increase in performance status lasting greater than 4 weeks from a baseline score of <70%, >50% reduction in morphine-equivalent analgesic consumption for 4 weeks from a baseline of >10 mg morphine equivalent per day, >50% improvement in pain scores from baseline >20 mm (visual analogue scale), with a secondary measure of >7% increase in weight sustained for >4 weeks. No CBR was assumed for patients who progressed within 4 weeks.

The aim was to recruit an initial 22 patients into the first stage of a two-stage Gehan design (based on 90% power and estimated 10% response rate), with the number of further patients recruited to stage two based on patient response in stage one. The trial was terminated at 19 patients following a presumed toxic death.

### Treatment

Irinotecan (a gift from Aventis) with atropine sulphate (300 *μ*g) prophylaxis (Gandia *et al*, 1993) was administered over 90 min following hydration (500 ml N/saline+20 mmol KCl+magnesium) over 30 min and cisplatin (25 mg m^−2^) administered over 30 min, on days 1 and 8 of a 21-day cycle. (The calculated dose intensity for irinotecan is approximately 50% of that utilised in single-agent Phase I studies, 46 mg m^−2^ week^−1^.) A maximum of five cycles could be administered (15 weeks), with weekly patient visits for clinical examination, toxicity evaluation, FBC, biochemistry, weight (prior to hydration), pain inventory and performance status assessment (worser of two scores determined independently by two observers). Loperamide and ciprofloxacin were provided for prophylaxis against irinotecan-induced delayed diarrhoea, as advised by the manufacturer. Chemotherapy was administered only if KPS⩾70%, neutrophils>1.5 × 10^9^/l and all other haematological and liver functions tests remained within normal limits. If either grade 3–4 diarrhoea or grade 4 neutropenia, or grade 3 neutropenia and infection occurred, then irinotecan dose was reduced to 35 mg m^−2^ (diarrhoea and neutropenia) and cisplatin reduced to 20 mg m^−2^ (neutropenia only) in all subsequent cycles. All other toxicities were recorded weekly, and any greater than grade 2 were treated with supportive care and a maximum delay in chemotherapy of 2 weeks. Continuous treatment with steroids was discouraged unless the patient had been on a constant maintenance dose for 2 weeks prior to trial entry, or there was persistent and severe loss of appetite following chemotherapy, severe liver capsular pain or there was chemotherapy-related delayed nausea and vomiting.

### Response and toxicity

Staging abdominal CT scans with contrast enhancement were performed within 2 weeks of the start and end of chemotherapy following a minimum of two cycles of treatment, and every 4–6 weeks thereafter, unless there was obvious clinical evidence of progression. The response evaluation criteria in solid tumours (RECIST) criteria were employed to immediately assess CT scans and to guide subsequent management, and all CT scans were again reviewed independently by one radiologist after closure of the study (Therasse *et al*, 2000). Toxicity was assessed after each treatment and graded using the National Cancer Institute of Canada Clinical Trials group (NCIC-CTG) expanded common toxicity criteria (CTC version 1).

## RESULTS

A total of 19 out of the 22 patients planned were recruited into this study from a single institution and analysed by intention to treat. One patient was excluded from response and toxicity analysis from the outset due to a rapid deterioration of a concurrent clinical condition that precluded consent to chemotherapy. Patient characteristics for the 18 remaining patients are shown in Table 1

**Table 1:** Patient characteristics at trial entry

	***N***	**(%)**
*Sex*		
Male	8	(44)
Female	10	(56)

*Karnofsky performance status*		
100	2	(11)
90	12	(67)
80	4	(22)

*Site of disease*		
Pancreatic head	15	(83)
Body and tail	3	(17)

*Stage of disease*		
IVA	6	(33)
IVB	12	(67)

*Differentiation*		
Well	2	(14)
Moderate	7	(50)
Poor	5	(36)

*Age*		
Median	61 years	
Range	38–74	

*Weight*		
Median	67.5 kg	
Range	42–93	

*Body mass index*		
Median	24.5	
Range	16.3–34.6	

*Diagnosis to entry*		
Median	26.5 days	
Range	9–258	

*Analgesic consumption (mg day^−1^ morphine equivalent)*		
Median	32.1 mg	
Range	0–120	

. In summary, the majority of patients had a KPS >90% at study entry (78%), metastatic disease (67%, stage IVB) from a pancreatic head primary (83%), and had not received previous chemotherapy or radiotherapy (one patient had previous immunisation against gastrin, which completed 6 weeks prior to study entry). One patient was taking steroids at entry, five patients had previous bypass gastro-jejunostomy and nine patients had concurrent medical conditions: ankylosing spondylitis+diabetes (1), bilateral deep venous thrombosis (1), diabetes (2), epilepsy (1), hypertension (3), controlled chronic schizophrenia (1). Delay between histological diagnosis and entry into the trial was approximately 4 weeks, but had a wide range. The first treatment was usually on the day of entry to the trial for 15 (83%) patients (two patients starting 4 and 7 days after entry and one consented patient did not receive treatment due to deterioration of performance status on the day of treatment). In all, 17 patients received a total of 89 treatments of combination chemotherapy between March 2000 and June 2001. Altogether, 75 (84%) of treatments were full dose (70 mg m^−2^ irinotecan, 25 mg m^−2^ cisplatin). In 14 cases, doses were reduced (14 doses to 35 mg m^−2^ irinotecan, 20 mg m^−2^ cisplatin) and six patients missed a total of 10 treatments because of toxicity. The actual mean dose intensity per patient of chemotherapy was 37.0 (range 17.5–46.7, median 40.8, protocol 46.7) mg m^−2^ week^−1^ for irinotecan and 13.7 (range 7.5–16.7, median 14.6, protocol 16.7) mg m^−2^ week^−1^ for cisplatin.

### Survival

In all, 15 of the 18 patients had died at the time of analysis. The three alive patients were censored in the survival analysis at 6, 6.5 and 16 months. The median overall survival was 5.0 (95% CI: 3.9, 10.1) months and median PFS was 3.1 (95% CI: 1.3, 3.7) months. All patients, but one, had stable disease or had progressed either radiologically or clinically within the 15 weeks study duration.

### Radiological response

Seven (39%) patients did not undergo post-treatment scans because of clinical evidence of progression (one patient with intrahepatic cholestasis from metastasis confirmed on ultrasound examination, four patients with a combination of rapid loss of weight, increased pain and rapid deterioration of performance status, one pulmonary embolism and one death). The remaining 11 patients had pre- and post-treatment CT scans with repeat post-treatment scans after at least 6 weeks. Using RECIST criteria, there were no complete responders, one partial response of low volume disease in pancreatic body and liver (PR=5%), three with stable disease, who were stage IVA (*n*=2) and IVB (*n*=1) (SD=17%, PR+SD=22%), and seven (39%) with progressive disease.

### Clinical benefit response (CBR)

Only one patient had a positive response to pain intensity (negative to analgesia), five patients had a positive/stable response to analgesia (all nonassessable for pain intensity), five patients were negative for both pain scores. The majority of patients were not eligible for KPS assessment, as their baseline performance status was above 70%, and were stable for weight, meaning that no CBR were detected using the strictly applied criteria of Rothenberg.

### Toxicity

Almost all grade 3 and 4 toxicity occurred within the first 4 weeks of treatment (Table 2

**Table 2:** Treatment related toxicity

**Reported toxicity episodes**	**No. of patients (max 18)**	**No. of doses (max 88)**
**CTC Grade**	***N* (%)**	***N* (%)**
Diarrhoea		
1/2	10 (56%)	17 (19%)
3	2 (11%)	1 (1%)
4	2 (11%)	4 (5%)

*Haematological*		
1/2	10 (56%)	28 (32%)
3	3 (17%)	3 (3%)
4	2 (11%)	2 (2%)

*Nausea*		
1/2	11 (61%)	23 (26%)
3	4 (22%)	4 (5%)
4	2 (11%)	2 (2%)

*Vomiting*		
1/2	7 (39%)	14 (16%)
3	2 (11%)	2 (2%)
4	3 (17%)	3 (3%)

*Other*		
1/2	11 (61%)	39 (44%)
3	1 (6%)	1 (1%)
4	1 (6%)	1 (1%)

Nontoxic SAE	3 (17%)	3 (3%)

). Of the 89 doses administered, five (6%) were associated with grade 3/4 diarrhoea, five (6%) with grade 3/4 haematological toxicity (neutropenia), six (7%) with grade 3/4 nausea and 5 (6%) with grade 3/4 vomiting.

The trial was stopped because of a serious adverse event classified as a toxic death, even though *postmortem* was refused: concurrent grade 4 diarrhoea, nausea, vomiting, pain and haematological toxicity, resulting in multiorgan failure and death. The patient had liver metastasis and a large head of pancreas primary encasing superior mesenteric vessels. Obstructive jaundice was slow to clear following biliary stent insertion prior to trial entry, although within normal limits on the day of chemotherapy administration. Emergency admission occurred after week 2 as a result of acute abdominal pain, hypotension and neutropenic fever. Neither the pain nor the fever responded to antibiotics, and the patient died of a presumed intra-abdominal catastrophic event, multiorgan failure and neutropenic sepsis.

Three additional serious adverse events were reported and all disease related (pulmonary embolus, deep venous thrombosis and gastrointestinal bleed after 1, 2 and 10 weeks (1, 2 and 7 doses) of treatment, respectively). Grade 3 and 4 neutropenia occurred during weeks 2–4 of treatment and were often associated with nausea and vomiting. In two patients, this correlated with slightly higher bilirubin levels in the normal range at entry into the study (not shown). This suggested that either intrahepatic cholestasis from metastasis or slow recovery from obstructive jaundice may have increased the half-life of Irinotecan metabolites (Sn38) and resulted in increased susceptibility to toxicity. ‘Other’ toxicities reported were 21, Grade 1 and 18, Grade 2 events: pain (5), alopecia (9), constipation (8), tiredness (4), appetite (1), oral (6), skin (1), pulmonary embolus and atrial fibrillation (1), transient raised creatinine (1), steatorrhoea (2), and upper respiratory tract infection (1).

## DISCUSSION

Our results show that the majority of patients with advanced pancreatic adenocarcinoma and good performance status can tolerate an irinotecan and cisplatin combination at modest doses, but that this combination appears not all that active, with response rates that are in line with other chemotherapy combinations in this disease. However, the disadvantage of this combination may be the severe toxicity in patients with advanced pancreatic cancer, as others have reported (Slater *et al*, 2002), which may be the result of either interindividual variability in drug metabolism or decreased biliary drainage from the liver following obstructive jaundice. Despite the small size of this study, we urge caution in the adoption of this combination in pancreatic cancer, even though some patients appeared to respond to treatment. Furthermore, recent reports also highlight the unpredictable toxicity that can occur with irinotecan using dosing based on body weight, which suggests that the use of a fixed dosing of this agent may be preferential (Mathijssen *et al*, 2002). If this combination were used again in advanced pancreatic cancer, we would dose irinotecan at a low level for at least the first cycle and judge dose escalation by nadir blood counts. We would also wish to obtain more information about the appropriate selection of patients prior to treatment (see below). Aside from unpredictable toxicity, we note that the haematological toxicity from this combination appears no different from single-agent irinotecan or combinations of irinotecan and gemcitabine (Wagener *et al*, 1995; Armand *et al*, 1996; Rocha Lima *et al*, 2002).

Radiological response rates and survival in this study are compatible with other single agent and combination treatments. Clinical benefit response criteria remain subjective and may be difficult to compare across studies depending on modified criteria, so we chose to follow the original criteria (Cascinu *et al*, 1996; Burris *et al*, 1997; Rocha Lima *et al*, 2002). With the modest trial selection parameters chosen here, almost all patients who tolerated full-course treatment had preserved weight and performance status, and controlled pain over the study period (not shown). While these patients are better at tolerating a 15-week chemotherapy course, they tended not to contribute to a CBR analysis, as strictly judged by the original published criteria of Rothenberg *et al* (1996b). The main reason for a lack of detectable benefit was the magnitude and duration of improvement from the baseline level at trial entry. Furthermore, the selection criteria for most trials lead to bias, as enrollment of patients with good performance status, with little pain and weight loss, are selected. Any change in CBR may be unrepresentative of the total population of patients presenting with advanced pancreatic cancer. A further selection bias may be related to the extent of disease, as patients with locoregional disease tend to have a prolonged survival (Bramhall *et al*, 2001). Thus, because we have adopted a Phase II approach with small numbers of patients, we cannot exclude a role for this combination in the treatment of a subgroup of patients that might tolerate treatment with minimal toxicity even at higher doses, and which may also have a higher response rate. One approach that might avoid continued reporting of negative Phase II studies such as this might be to attempt to optimise current combination therapy to clinical subgroups of patients with advanced pancreatic adenocarcinoma. For example, good performance status patients with locoregional disease may tolerate high-dose combination treatments and gain most palliative benefit from their use. The assessment of palliative benefit *vs* toxicity for new combinations of chemotherapy and biological therapy in pancreatic cancer may require the stratification of patients in future phase II trials.
